# From Conservation to Connection: Exploring the Role of Nativeness in Shaping People’s Relationships with Urban Trees

**DOI:** 10.1007/s00267-023-01856-3

**Published:** 2023-07-15

**Authors:** Haylee Kaplan, Vishnu Prahalad, Dave Kendal

**Affiliations:** Healthy Landscapes Research Group, School of Geography, Planning, and Spatial Science, University of Tasmania, Hobart, TAS Australia

**Keywords:** Urban greening, Human dimensions, Semi-structured interviews, Plant choice, Exotic, Provenance

## Abstract

Deciding whether to plant native or non-native trees in public urban green spaces is becoming complex and conflicted, and decisions purely based on biotic nativeness are likely to be hamstrung as climate change and rising urban heat push many native species beyond their natural ranges. Importantly, tree selection considerations by urban planners and environmental managers will have to move beyond a primary focus on securing conservation and ecological outcomes, to elucidate and engage with a growing interest in the socio-cultural values and services of urban trees. Building on emerging theoretical perspectives, this place-based study explores the role that perceptions of nativeness have in shaping people’s relationships with native and non-native urban trees and landscapes in an Australian city. Nativeness was associated with a range of subjective meanings including cultural identity, political expression, nature connection, desirable and undesirable traits, and environmental and cultural compatibility. Our findings emphasise that the ways in which urban trees and green spaces are valued and experienced is likely mediated by people’s perceptions of nativeness and its importance relative to other attributes. To provision and sustain green spaces that meet the diverse needs and preferences of urban publics, planners and managers need to elucidate and incorporate the nuanced, place-based and multifaceted subjective meanings of nativeness into urban greening decision-making and practice.

## Introduction

Maintaining healthy urban forests is a key sustainability challenge for cities around the world, particularly in the face of increasing climate stressors (Kendal et al. 2018). Urban trees are vital for the health and wellbeing of humans and wildlife in cities, providing a broad range of benefits such as microclimate regulation, habitat provision, and aesthetics (Elmqvist et al. 2015). While many factors have driven the selection of urban tree species, a highly cited attribute, and a most contentious aspect of urban forest management, involves the ‘nativeness’ of public trees (Kirkpatrick et al. 2013, Conway and Vander Vecht 2015). Even though nativeness is a widely used ecological term, it is a socially constructed concept and, in urban contexts, often arbitrarily determined (Berthon et al. 2021). There is a need for a clearer understanding of public views surrounding the nativeness of urban trees to inform debates around their use and management.

The concept of nativeness, referring to the biogeographic status of species (Essl et al. 2018), and its use to evaluate species in a given ecosystem has been the subject of vigorous and often polarised debate (Davis et al. 2011; Simberloff 2011). The prevailing approach in environmental management revolves around safeguarding native species while eradicating or controlling non-native species, reflecting traditional conservationist values (Lennon 2017). Nevertheless, numerous authors challenge this perspective and advocate for recognizing non-native species as an inevitable aspect of our globalised and extensively transformed world, highlighting their positive contributions to biodiversity and other ecosystem services (Hill and Hadly 2018, Schlaepfer 2018). This has broad implications for decision-making and resource allocation for species conservation and management of environments. Moreover, many have questioned the validity of the concept of nativeness itself as a dichotomous means of classifying species and indeed the potentially harmful socio-political connotations that it invokes (Peretti 1998; O’Brien 2006; Warren 2007).

Nevertheless, nativeness remains central to debates around ecological integrity and ecosystem functioning in urban green spaces (Conway et al. 2019). Although it does not always hold up to scrutiny, nativeness is often used as a proxy for environmental compatibility when selecting species for urban landscapes (Kendle and Rose 2000). For instance, native plants are often assumed to be functionally better adapted to local environmental conditions, however this may not always be the case, particularly in highly modified novel urban environments to which non-native trees may be more tolerant (de Carvalho et al. 2022). Indeed, the novel ecosystem approach (Hobbs et al. 2009), which re-evaluates the role of non-native species as integral to the functioning of many anthropogenically altered ecosystems, has shifted the paradigm in urban ecosystem management towards a more adaptive approach to native and non-native tree selection and management (Kowarik 2011).

This is not to say that conserving native species in cities for their biodiversity values is not important (Ives et al. 2016), but in the context of public trees, exclusively prioritising native trees over non-native ones often neglects the many social values and functions provided by non-native trees in urban forests. In many instances, non-native trees are more readily available in the horticultural trade (Almas and Conway 2016; Pincetl et al. 2013) and through cultivation are often better able to thrive in urban conditions, thus representing a more sustainable option for ecosystem service provision (Schlaepfer et al. 2020). Ultimately, the primary functions of urban nature are human-centred (e.g. seasonal shading, aesthetics, food production, cultural representation), and people’s values and preferences must be incorporated into urban greening decision-making. As urban greening decisions need to engage with place-based social expectations in a changing climate (de Kleyn et al. 2020; Jim and Chen 2006), it is important to incorporate the social and cultural dimensions of ‘nativeness’ of public trees to reflect the values and preferences of urban publics.

Yet, our understanding of the human dimensions of nativeness in urban green spaces is relatively limited (Kaplan et al. 2021), with the bulk of current research on urban tree nativeness focused on ecological dimensions of urban nature, such as biodiversity, functional traits, and ecological resilience. For instance, we know that in general, having more native plants in cities may be beneficial for supporting native animal species, but these effects may depend more on the resources a plant provides than its origin (Berthon et al. 2021). We also know that the physiological traits of native plants can constrain their abilities to use resources in highly modified urban environments and their adaptive capacity to climate change (de Carvalho et al. 2022). Moreover, there is an abundance of literature on the value, performance, and biogeography of non-native plants in non-urban settings (e.g. Castro-Díez et al. 2019, Schlaepfer et al. 2011, van Kleunen et al. 2015). However, despite much research and discussion on the relative merits of native and non-native trees, there is relatively little evidence elicited on how the concept of nativeness actually affects people’s relationships and interactions with urban public trees. This is despite many local governance bodies being responsible for provisioning key services (including a growing range of cultural services; Fish et al. 2016) relying on public support in their decision making on urban trees.

Notwithstanding the ambiguities in the technical definition of nativeness (Warren 2007), people’s perceptions are often complex, drawing from pluralistic subjective meanings when forming opinions on native or non-native species (Kaplan et al. 2021). Unsurprisingly, research on urban public perceptions of nativeness has found highly varied, in some cases polarised, opinions on native and non-native species. For example, studies involving Australian residential gardeners (Zagorski et al. 2004; Head and Muir 2006; Kendal et al. 2008) have noted opposing attitudes and preferences towards nativeness in garden plants. Such disparate perceptions of nativeness may lead to conflicts around urban greening practices and policies. Navigating these conflicts will likely become increasingly relevant as climate change in combination with urban heat and other urban stressors is predicted to make the ongoing use of native plants unsustainable in many cities (Kendal et al. 2018). For instance, in Australia, more than two-thirds of urban plants face considerable reductions in climatically suitable habitat over the coming decades (Kendal et al. 2017; Burley et al. 2019). Responses to these challenges may include introducing more climate-adapted (non-native, or at least not locally native) species into the urban forest to enhance resilience (Sjöman et al. 2016). Cities may soon see a shift toward these more novel approaches to urban greening as they become more acceptable to practitioners (Teixeira et al. 2022) and the public (Hoyle et al. 2017), as changing physical and social realities weigh in on urban greening strategies.

While we have a climatological basis for predicting the biodiversity implications of a shift in urban forests away from native trees, there is less clarity around the impacts such adaptations will have on urban residents whose experiences of public trees often form a meaningful part of everyday life (Pearce et al. 2015; Tansil et al. 2022). Most of the research on people’s perceptions of nativeness to date has been conducted in private residential gardens (but see Hoyle et al. 2017; Noe et al. 2021) where individual preferences drive plant choices. There is an important gap in understanding the meaning of nativeness in public spaces where people have less direct influence on the landscape, and conflicting values of different parts of the community will require trade-offs in decision-making. Understanding how and why people evaluate urban trees and landscapes based on their nativeness is a crucial first step in facilitating such trade-offs. This can help shed light on ways that urban greening strategies can create green spaces across cities that provide opportunities for diverse urban populations.

Previous research in Australian cities (Head and Muir 2006; Trigger and Head 2010) has highlighted the relational nature of people’s interactions with native and non-native urban nature. In their ethnography of Australian suburban gardeners, Head and Muir (2006) show how people’s preferences for garden plants stem from their views on nativeness, which ranged from strongly nativist to strongly antipathic to native plants. These relational values and preferences for native and non-native species are shown through their work to be culturally formed and dependent on the meanings given to nativeness by individuals.

In this study, we aim to expand our current understanding of the subjective meanings of nativeness and how these may shape people’s experiences of urban nature by focussing on public urban green spaces (e.g. public gardens, parks, urban reserves, or tree-lined urban streets). Drawing from environmental psychology and human geography, we adopt a broad understanding of ‘meanings’ as constituting the ideas, beliefs, and values that help people to make sense of biophysical landscapes (Williams and Patterson 1999). In other words, meanings are the subjective interpretations and connotations that people ascribe to features in the environment through their experiences and social interactions. Using a place-based, qualitative approach, we address the following research questions: *(1) What meanings and values do people ascribe to the nativeness of urban trees and landscapes*, and (*2) how do subjective meanings of nativeness shape people’s experiences of urban green spaces?* The findings of this study are intended firstly, to strengthen and expand on current theoretical perspectives on social perceptions of nativeness in urban contexts, and secondly to help inform management planning and decision-making around native and non-native trees in urban greening practice and policy.

## Methods

We used a qualitative approach to address our research questions, employing semi-structured interviews with members of the public from our study location. The choice of our method was to enable us to develop a rich and nuanced understanding of how people attached meanings to their experiences of and relationships with public trees in urban landscapes. As our intention was to elicit these place-based meaning in the context of our study area, the interviews were expected to help understand how trees (and their provenance) help create meaning within certain places. The protocols used in this study were approved by the Tasmanian Social Science Human Ethics Committee (S0022983).

### Study Location

This study was conducted in Hobart, the capital city of Tasmania, Australia. With a population of around 247,000, Hobart is a small but rapidly growing city, with nearly two-thirds of net population growth between 2015–2021 due to international immigration (ABS Census data 2022). Culturally, the majority (77%) of Hobart’s population is of British/Irish descent, and 4% are Australian Aboriginals and/or Torres Strait Islanders (ABS Census data 2022). Geographically, Hobart is unlike most other Australian capital cities in its overall proximity to large expanses of remnant bushland. Bushland reserves, including Wellington Park, one of Tasmania’s large, protected areas, are accessible within 10 km from the city centre. This accessibility to bushland reserves means that many Hobart residents have frequent exposure to peri-urban native landscapes in addition to more typical urban green spaces like parks, gardens, and street trees. Within the city itself, public urban green spaces are more limited and occupy only around 17% of Hobart’s urban areas, which is comparable to other urban centres in Australia (Hsu et al. 2022). Similar to other Australian capital cities, the City of Hobart’s urban greening strategy aims to increase public tree canopy cover to 40% over the coming decades using a mix of native and non-native species (City of Hobart 2017).

### Recruitment

The population of interest was people over the age of 18 years living in an urban or suburban area in Hobart. The aim of our recruitment strategy was to capture a diverse range of views in our population of interest, until concept saturation was reached. In this process, we did not purposively seek socio-demographic representativeness in our sample due to both ethical considerations and its likely tangential value in addressing our research questions within the scope of this study. Recruitment was conducted via online advertisements posted on social media. To recruit participants with potentially different views on nativeness, we advertised in both gardening and nature-based interest groups on Facebook. Each group represented a particular orientation or ethos relating to plants in cities (Table 1): Ornamental gardening, permaculture, wildlife gardening, native plant enthusiasts, weed spotters. We also advertised on two general community Facebook pages that did not have any specific nature-based orientation. To reduce potential bias in the sample, we chose to omit any mention of nativeness in the advertisement to avoid oversampling people with strong views on nativeness.

**Table 1:** Groups targeted for recruitment of interview participants

Target group	Orientation	Example social media groups
Ornamental gardeners	Appreciation and enjoyment of ornamental garden plants (with emphasis on non-native horticulture species)	Gardening in Tasmania
Permaculturalists	Landscape design guided by environmentally conscious and community-oriented urban agriculture principles	Permaculture Tasmania
Native plant enthusiasts	Appreciation and enjoyment of native plant species predominantly in natural settings	Tasmanian Native Flora
Wildlife gardeners	Landscape design intended to support urban wildlife predominantly through use of native horticulture species	Gardens for Wildlife Tasmania
Weed spotters	Detection and identification of new or potentially invasive non-native species in natural and urban settings	Tasmanian Weeds
General community	Community-oriented activities such as events or sports	Kingborough Community Group

### Interviews

Semi-structured interviews were conducted by the lead author (HK) between October 2020 and May 2021. Interviews lasted approximately 1 h and took place at various locations chosen by the participants. Interviews were conversational and interview prompts (see Appendix A) were used to guide the conversation. Interview prompts were designed (following Kaplan et al. 2021) to give participants the opportunity to explore their own values and meanings around nativeness, including the benefits and risks that they associate with native and non-native trees in public urban spaces.

Participants were first asked about their everyday experiences visiting green spaces in the city, and their awareness of the native and non-native trees in those spaces. Definitions of ‘native’ and ‘non-native’ were not provided to participants; instead, they were asked to describe their conceptions of what makes a plant native and non-native. They were then prompted to talk about how and why native and non-native trees were important to them in the urban green spaces they visited, and whether they believed that native and non-native species belonged in Hobart. Participants were lastly asked to think more generally about the problems and benefits they associated with native and non-native trees in the city. Interviews were audio-recorded and reflexive notes were taken after each interview to aid the analyses.

### Analyses

Audio recordings of the interviews were transcribed verbatim, and transcripts were coded and thematically analysed using NVivo software (release 1.2, May 2020; QSR International 2020). We used an inductive approach to the thematic analysis (Braun and Clarke 2012), allowing codes and themes to be derived directly from the data. After familiarisation with the transcripts, meaningful segments of data were extracted and given initial descriptive codes based on semantic or latent meanings. Initial coding was conducted by the first author (HK) and verified by the other two authors (DK, VP). Codes were then iteratively compared, mapped, and reviewed to develop themes and sub-themes that capture important elements in the data. Themes were developed and refined consensually among all three authors at face-to-face meetings to ensure consistency in the interpretation of the data.

## Results

A total of 20 participants aged between 20 and 72 years were interviewed. Twelve of the participants were aged 60 years or older. Fourteen participants were female, 6 were male. Most of the participants were born in Australia, however 5 participants were born in other (European) countries and had moved to Australia in childhood or as adults. The following section provides a brief overview of the participants’ general perceptions of nativeness that may be underlying drivers of the meanings they ascribe to Hobarts’ native and non-native trees. This is followed by the findings from the thematic analysis.

Firstly, participants varied in the depth and precision of their conceptions of nativeness. Most participants included a spatial component when defining nativeness, and while for many this simply meant differentiating between Australian and non-Australian species, other participants adopted multiscalar definitions of ‘native’ (from locally endemic to Tasmanian native to Australian native). This seemed to be particularly useful for the Tasmanian context, being biogeographically separate from mainland Australia. These ‘levels’ of nativeness were assigned different weightings; locally endemic was seen as more intrinsically valuable than species broadly native to the continent (of mainland Australia). Some participants’ conceptions also included a temporal component, and European colonial settlement since 1788 was often cited as a pre-European reference point for non-native introductions to Australia. But nativeness, at least at the local Tasmanian scale, was seen by some participants as dynamic and subject to change over time. For instance, one participant described a category of unofficial native species as

“the things that don’t come from Australia, but they have been accepted. They’ve got their passport… In Tasmania, even though it’s a declared weed, hawthorn often gets accepted like that because it’s part of our Tasmanian landscape… it’s a native because it’s been here for 200 years” [06].

Another participant also spoke to this acceptance, but in the context of climate change, that introduced species be ‘allowed’ to be adopted as native.

Participants’ perceptions could be further differentiated based on two factors: nativism and salience. We noted that participants’ attitudes fell along a spectrum from strongly nativist (i.e. strong preference for native species, viewing non-native species with antipathy or suspicion) to ambivalent about nativeness (i.e. not averse to non-native species, but still tend to view native species as more intrinsically valuable). Notably, we did not discern in our sample any antipathic attitudes towards native species. In general, participants whose attitudes leaned towards the ‘nativist’ end of the spectrum also tended to have more precision and complexity to their conceptions of nativeness.

Additionally, nativeness had varying levels of salience to participants. By ‘salience’ we refer to the degree to which nativeness informs the ways in which people think and gather information about nature and their decision-making and behaviours in relation to species and landscapes (Miller et al. 2016). For participants with greater salience, nativeness was more likely to play a role in their reasons for visiting different green spaces and they had more explicit knowledge about nativeness. Whereas other participants with low salience of nativeness had more tacit understandings of nativeness and their reasoning for visiting green spaces did not tend to be motivated by the presence of native or non-native species.

### Themes identified

Participants associated a wide range of meanings with nativeness in Hobart’s green spaces. Thematic analysis categorised these into four central themes: Identity, Nature experience, Desirable and undesirable traits, and Urban compatibility, with 10 sub-themes. Table 2 provides a summary of the themes and sub-themes including the meanings associated with native and non-native trees respectively. Central themes should not be construed as mutually exclusive categories; there were overlapping and intersecting meanings across all themes and sub-themes. A detailed examination of the themes is presented below. Themes and sub-themes are highlighted in **bold** text and illustrative excerpts from the transcripts (edited for clarity where necessary) are shown in *italicised.*

**Table 2:** Meanings associated with native and non-native trees in Hobart green spaces across four central themes derived from public participant interviews

Central theme *Sub-theme*	Native trees	Non-native trees
**Identity**		
*Personal attachment*	Familiarity Feeling of ‘home’, belonging	
*Cultural identity*	Pride in unique local flora Cultural heritage	Loss of cultural identity
*Expression of political ideology*	Decolonisation	Reminders of colonial past
**Nature experience**		
*Nature connection*	Feeling connected to nature Improving wellbeing	Feeling disconnected from nature
*Cue to protect nature*	Authentic nature In need of human protection	Native species loss
*Understanding nature*	Appreciating local nature	Appreciating global nature
**Desirable and undesirable traits**		
*Amenity*		Aesthetics and variety Greenness/leafiness Cooling and shelter Seasonal awareness Urban agriculture
*Problematic traits*	Fire hazard Falling/dropping limbs	Invasiveness Requires additional maintenance
**Urban suitability**		
*Environmental suitability*	Suited to less modified environments	Tolerant of modified urban conditions
*Cultural context*	Evoke historical events (e.g. Australian Federation)	Memorialize historical events (e.g. major wars) Compatible with older architecture

### Theme 1: Nativeness and identity

When reflecting on the value of native plants in urban green spaces, most participants, particularly those who had grown up in Australia, spoke about Australian native species as an important part of their identity.

Several participants expressed their strong **personal attachment** to native trees and landscapes which provided a sense of ‘home’ and familiarity. One participant described being reminded of her connection to home while travelling outside of Australia:

“When I saw gum trees and I could smell gum trees and I just thought it just brings you back to being home. So yeah, it’s part of who you are, how you’ve grown up.” [15].

This was similarly the case for people not originally from Australia. Two of the participants who had immigrated from Europe found European trees planted around the city to be reminiscent of their childhoods and home countries:

“I just like the smell of pine trees. I like the atmosphere of pine trees… I was born in the Black Forest in Germany. So maybe it reminds me of that… I have an attachment to that kind of European forest” [20].

Native flora were also seen as symbols of **cultural identity**, for example being Australian or Tasmanian, and the uniqueness of local native species often invoked a sense of pride and reverence. As one participant put simply, “*I really love these plants and this environment that is uniquely ours*” [19]. In the context of Hobart’s green spaces, having native species in urban parks and reserves was seen as important for retaining accessible links to this cultural heritage. Indeed, some participants expressed concerns that non-native plants in the city risked a loss or disconnection with this identity.

“Like the eucalypts and things. [It’s] home, it’s ‘Ah, that’s Australian, that’s my identity’… you don’t want those lines to get really blurred, or plants that were introduced to be considered native one day, I guess, because then we’ve lost a bit of that identity.” [19].

Conversely, non-native plants were viewed unfavourably by some participants as symbols of European colonial history and heritage.

“To cultivate and appreciate native plants is to engage with [or] accept where we are and where we’ve come from. So, I see the more exotic plantings as a constant affirmation of our White settlement history, our colonial heritage. So, I think there’s a great challenge in shifting that.” [17].

Linked to the above, planting natives around the city was considered an important **expression of political ideology**, particularly relating to decolonisation and restitution for the harms done to Aboriginal peoples by European settlers and their descendants. Planting native species in the city was seen as a means to normalise these beliefs in broader society.

“I think decolonisation is really important and I think we should be listening more to Aboriginal and Torres Strait Islander people. And, I mean, I can’t speak for anyone, but I feel like prioritising native plants would be something that would be valuable to them…. And I guess if we prioritised native plants, then that would kind of show a change in attitude as to what is important and hopefully would put a little bit more value onto things like that.” [20].

### Theme 2: Nativeness and nature experience

A second key theme that emerged was that nativeness influenced the ways that participants felt about and experienced nature in green spaces. Native species and native landscapes in cities were important to participants who sought **connection to nature**, i.e. a sense of affinity to the natural world and having a relationship with fauna, flora, and natural landscapes. For some participants, the nativeness of the species in a landscape influenced the sense of connection to nature they felt. One participant said that she visited native landscapes in Hobart to get a “*dose of natives*” for her wellbeing:

“I get enjoyment from it. I feel relaxed. I feel like I’m connected to the place that I am in… I would specifically go somewhere because it’s full of native plants.” [01].

Although it was not stated explicitly, many responses implied that native species represented a more authentic nature that was distinct from urban landscapes. One participant alluded to this when saying that “*[native plants] bring nature into the city. And some people never see those things because they don’t go out of the city*” [15], implying that non-native trees and novel ecosystems in cities are not ‘true’ nature. Indeed, several participants noted their concerns that non-native species in the city might be causing a disconnection with nature and that that would have broader ramifications for society and the environment.

“I feel that people are quite disconnected from the natural environment, particularly in cities, and that sort of disconnection means that we just keep consuming. So that means that we end up destroying more of those natural areas and ultimately, we’re going to mess things up for ourselves and all the other species in the world.” [01].

There was a strong belief among most participants that native species had an intrinsic right to exist, and nativeness served as a **cue to protect nature** in the city. One participant described the loss of native species in cities as “evil” and stressed the need for actively planting and maintaining native plants in urban green spaces: “*So you need to keep [native plants] in the parks to keep them close to their original habitats… Particularly when they’re important for other things to survive. Besides which, they have a right to exist* sui generis.” [06].

These participants were also aware of the potential ecological knock-on effects of native plant species loss for urban wildlife, as well as the implications for humans.

“We have lost too many of our native animals anyway. We invade their space, push them out, kill them, make them move on. I just don’t see that it’s fair. And I think with any kind of chain with plants, bugs, animals, anything, you know, once you start losing them, it’s a breakdown of everything. It’s risking your own future as well. Very basically because that was their home first and we’ve come in and destroyed it.” [08].

For many participants, nativeness provided a crucial lens to better **understand nature**. Knowledge of which species are native and non-native in urban green spaces was seen as an important element of appreciating and protecting nature. Not only were some participants particularly eager to distinguish native and non-native species, but they also stressed the need for the public to have the same awareness.

“I think people aren’t aware of what’s native and what’s not native. So, I think it’s important that people are made aware of what’s native and what’s not. I think it’s important for our environment and for the future of our greenery, if you like, that people are aware that some of the things we think are native aren’t. And that people are aware of what is so that they can be protected in the long run” [14].

However, non-native species were also considered important in urban green spaces for providing opportunities to learn about nature in other parts of the world.

“It’s education, to go have a look and see what other plants are like. We don’t want to shut ourselves in a box and think that the bush around us is the only bush there is. In different parts of the world there’s different bush. Good to see it.” [13].

### Theme 3: Desirable and undesirable traits

Participants were aware that urban nature was functional, and most agreed that a mix of native and non-native trees was needed to serve many of the desired human-centred functions and services in the city. Non-native species were perceived to have greater **amenity value**. Aesthetically, non-native plants were perceived to add colour and variety to the urban palette. Non-native trees were generally considered to be more vibrant and attractive (to people) than native species. Participants highlighted the greenness and leafiness that certain non-native trees create in Hobart’s urban environment which native trees could not.

“It’s like an amenity and that’s what Australians I feel often are attracted to. I don’t know that you can always create with Australian natives the same sort of lushness or big grassy spaces. So, non-natives in those settings create a space or an atmosphere that people enjoy being in and using.” [01].

Large spreading non-native trees in particular were valued for providing shade and shelter. This characteristic was perceived to be lacking among native trees.

“[Oak trees] in a public park, they’re great, they spread out, they’re spectacular, they’re a place to shelter under when it rains. And they make a park look park-like… We don’t have native spreading trees.” [06].

Similarly, deciduousness in non-native plants was deemed to be desirable for urban temperature control, by providing cooling shade in warmer months and allowing the sun through to warm surfaces in the cooler months. Moreover, the colour-changing foliage of non-native deciduous trees was valued for creating awareness of the changing seasons.

“[Having non-native deciduous trees] does help our awareness of changing seasons. We know they happen, but if just left to our native [trees] we probably wouldn’t notice it so much. But it gives an added dimension of awareness and a reflection of life, of changing seasons.” [07].

Non-native plants were also valued for their function in urban agriculture. Several participants wanted to see more edible fruiting trees in urban green spaces and noted that native species were generally not suited to this purpose.

However, participants commented on a number of **problematic traits** (or disservices) that they associated mainly with non-native trees. “Weedy” traits, such as the ability to disperse uncontrollably and displace native species, were mentioned often, particularly by participants with strong nativist beliefs. Similarly, traits that could potentially cause damage to infrastructure and waterways, such as invasive roots or dropping large amounts of foliage or fruit, were more strongly associated with non-native trees. Although these traits were often linked to specific species that are known to be invasive, such as cotoneaster (*Cotoneaster* spp.*)*, willow (*Salix* spp.), or gorse (*Ulex europaeus*), in general non-native species were often viewed with greater suspicion than native species in this regard. Lastly, further concerns were raised about the perceived higher maintenance needs of non-native trees, such as additional fertilizer or pruning that requires more resources and may have detrimental flow-on effects to the environment.

### Theme 4: Urban compatibility

When asked where native and non-native species belong in Hobart, only two participants felt strongly that the city should contain native species exclusively.

“[If] we can revert to our native species providing shade and shelter and everything else, I think that’s the way to go. I don’t see the purpose in putting a non-native species in the ground in a country that it doesn’t belong in” [04].

However, for most participants the answer was context dependent. Firstly, participants generally considered the **environmental compatibility** of nativeness. They were aware that urban environments are not always ideal conditions for native species to survive, due to their human-modified abiotic conditions. Non-native species were generally considered more acceptable for these highly modified urban landscapes, firstly because the conditions were deemed unfavourable to native species and secondly, because the urban environment was perceived to be less natural:

“I think anywhere that’s already been disturbed by human activity could probably do with non-native species. It’s too hard to recreate what’s been lost, so as long as it’s useful or beneficial in the short and long term, I’m not opposed to non-natives. Especially along streets and things like that.” [03].

Although one participant held an opposing view that native species were more tolerant to harsher conditions and that non-native species were only surviving in cities due to being ‘*pampered*’ [10].

A second component of environmental suitability involved the risk that native trees posed to human safety and infrastructure. Gum trees (*Eucalyptus* spp.), which are the dominant native tall canopy tree species in Tasmania, were brought up often by participants when considering the risks of planting native species in the city. A common perception was that gum trees are prone to falling and dropping large limbs. As one participant put it: *‘That makes* [gum trees] *spectacularly bad for cities.’* [06]. Additionally, a greater bushfire risk was often associated with native trees, particularly gum trees. This led participants to consider certain parts of the city where these risks were more consequential, such as in streets and gardens, unsuitable for some native trees.

Secondly, some participants felt that the **cultural context** of places in the city should signify their suitability for native or non-native trees. Native and non-native trees were seen to signify important historical events such as European settlement, Australian Federation, or commemorations of major wars. The historical significance of trees in Hobart, particularly European trees such as oaks and elms, granted them special status of suitability and belongingness in Hobart’s older green spaces.

‘They [non-native trees] are part of the land, I guess, because it is part of the heritage in the landscape, which was why they were planted in the first place’ [07].

Similarly, older styles of architecture which are common in the city centre and older Hobart suburbs were also considered to be better suited to non-native species. Some participants felt that as Hobart becomes more multicultural, new mixes of native and non-native trees could be added to the landscape to represent this. “*We’re multicultural, so people like to see* [species] *that remind them of other places. I’d love to see jacarandas.”* [11] One participant described what she saw as a limited selection of street tree species in Hobart as “*a kind of an unenlightened cultural oppression*.” [17].

## Discussion

### Summary of key findings

To deepen our understanding of the social values of urban trees and how this might affect people’s experience of urban greenspaces, this study set out to explore the subjective meanings that people attach to the nativeness of trees and landscapes in urban contexts. In contrast to the ecological sciences where the terms ‘native’ and ‘non-native’ hold strict (albeit contested) scientific meanings, among the general public nativeness connotes an array of intersecting and sometimes conflicting meanings. Our study found four clusters of meanings that people ascribe to the nativeness of urban plants:

1. Identity (species native to one’s home represent a sense of belonging, familiarity, and cultural and political identity);
2. Nature experience (native status influences one’s connection to and appreciation of nature);
3. Desirable and undesirable traits (novel attributes of non-native plants contribute both amenity values and ‘weedy’ traits to green spaces); and
4. Urban suitability (the perceived appropriateness of native and non-native plants in different urban environmental and cultural contexts).

Evidently, and perhaps unsurprisingly, the meanings of nativeness held by the public are not always consistent with ecological concepts of nativeness. People’s subjective views of nativeness are much broader and more heterogenous than the rigorously defined ecological concepts of autochthony, naturalisation, or invasiveness. This is precisely at the root of the challenges that urban planners and environmental managers increasingly face in seeking to manage urban greenspaces to meet public preferences amidst a changing climate. An ‘only native species’ in cities approach, motivated by a limited set of perceptions, is inconsistent with both community views and a growing body of literature that argues for a multifunctional approach that is fit for purpose in novel urban settings (Kowarik 2011; Teixeira et al. 2022). A starting point to overcome this dissensus is to seek to explore community views, as our work has sought to do, and use these themes as part of negotiating practical, equitable, and socially acceptable management outcomes through collaborative and deliberative planning processes involving expert, governmental, and community stakeholders (Berkes 2009).

We recognise that the themes that emerged from this study are largely consistent with the findings of previous qualitative research on perceptions of nativeness. A recent review of social research on nativeness (Kaplan et al. 2021) showed that people’s meanings surrounding nativeness generally fall along 5 dimensions (Belonging, Amenity, Human influence, Negative impacts, Environmental compatibility, and Identity), similar to those we identified in the present study (see Table 2). However, there was one notable exception of the meanings surrounding Nature experience, specifically the role of nativeness in people’s nature connection, and how they learn about and understand nature (both consciously and unconsciously). The perception that native trees and landscapes enhance human-nature connection in cities has not received attention in the literature, as far as we are aware (also see Kaplan et al. 2021). However, one quantitative Australian study found that people with greater nature relatedness were more likely to seek out green spaces with remnant native vegetation (Shanahan et al. 2015). Equally, we have found little previous research exploring the educational benefits of nativeness in urban trees in understanding local and global nature (but see Sosa et al. 2021). Together with nature connectedness, environmental knowledge is a key driver of pro-environmental behaviour (Otto and Pensini 2017). In our study, both native and non-native trees promoted environmental knowledge and nature appreciation. We therefore see this as a promising area for further development of theory.

Altogether, our findings show that the ways that people value nativeness are nuanced and need to be contextualised by cultural, environmental, and historical factors. Our results also point to two underlying factors that may drive people’s attitudes and behaviours towards nativeness. Firstly, nativeness was defined and interpreted in several ways that reflected not only people’s level of understanding of the concept, but also their conception of (urban) nature. For instance, while some people viewed nativeness as a transient status, subject to change over time or due to shifts in climate, to others nativeness was seen as equivalent to a fixed trait. Similar findings have been reported in other studies on urban landscapes (Hoyle et al. 2017) and invasive species (Schüttler et al. 2011). This has implications for how people might respond to changes in the nativeness of public urban flora. For example, introductions of new tree provenances may be less acceptable to people for whom ‘native’ denotes local provenance species than to others, to whom it may comprise any Australian or climate-adapted species.

Secondly, and another key finding from this study, the salience of nativeness varies between individuals. Salience is a key determinant of action (in support or opposition) in relation to public issues (Crawley et al. 2019). In the context of urban greening, people for whom nativeness has high salience may be more disposed to protest or alter their visitation behaviours in response to changes in the nativeness of urban green spaces. Previous research has documented the disconnection between people’s values and beliefs around nativeness and their behaviours, for example valuing native plants but choosing to maintain an exotic garden (Uren et al. 2015; Noe et al. 2021). One reason for this disconnect may be that nativeness is a less salient aspect of some people’s decision-making relative to other aspects, such as growing food or aesthetic enjoyment. We therefore suspect that salience may be an important mediator of behaviour regarding nativeness, however salience remains an underexplored factor in the social science literature on nativeness. Further research is needed to better understand the relationship between salience and other cognitive antecedents of behaviour towards native and non-native species. This research can also be extended to understanding the socio-demographic characteristics, including First Nations peoples, that might influence public perceptions and the importance they place on nativeness in urban greenspaces.

### Perceived nativeness and experiences of urban green spaces

Our findings also reveal that the nativeness of urban trees influences the ways in which many people use and experience urban green spaces. Some of the perceived benefits of native or non-native urban trees are linked to traits with tangible benefits (Andersson and McPhearson 2018); attributes such as dense, spreading canopies or production of edible fruit are found in non-native trees in Hobart and these can allow green space users shade or foraging opportunities, respectively. However, in other instances, the ways in which native or non-native trees or landscapes are used may be largely mediated by the intangible meanings that people give to them. For example, for some of the participants in our study native landscapes provided a sense of wellbeing and connection with nature. While several studies have shown that greater exposure to green spaces in cities facilitates wellbeing benefits (Jimenez et al. 2021), it has to be observed that there is no clear link to any tangible attributes unique to the native species in those landscapes compared with non-native species. Instead, some people subjectively perceive native trees and landscapes to be more ‘natural’ which may enable those green spaces to produce wellbeing benefits (Samus et al. 2022). Similarly, native species can likely only generate a sense of cultural identity for those who associate those meanings with nativeness. This may be a source of conflict or exclusion for some groups, such as migrants or people with differing political views. In terms of educational value, both native and non-native trees gave people opportunities to learn about nature through recognition and knowledge of local species and comparisons with species from other parts of the world.

### A shift in social values?

Interestingly, our findings may signal a shift in the way that Australian native plants are valued, at least in public spaces. Although we did find considerable variation in values assigned to native and non-native species, in contrast to previous research in Australian cities (Zagorski et al. 2004, Head and Muir 2006), we did not discern any strong preferences for non-native trees nor antipathy towards native trees. Furthermore, strongly nativist views were only represented in a small minority of the participants and most participants valued both native and non-native species for a range of different reasons. This may suggest a convergence towards a more nuanced consensus position on nativeness. While this may also be attributable to a potential lack of representation in our sample, similar findings were reported in a recent New Zealand study on urban plants (Noe et al. 2021). As with other post-colonial nations, nativeness is intrinsically linked with Australian cultural identity and national imaginaries (Smith 2011). Social norms around the use of native species in Australia have changed over the last century in response to cultural and political changes (Trigger and Mulcock 2005; Dyson 2016). In recent decades, much of this change has been towards popularising the use of local native species for their biodiversity benefits or to mitigate risks of biological invasions (Zagorski et al. 2004; Shaw et al. 2017). It is likely that now there is a more nuanced understanding of the role and place of natives potentially following the development and use of the concept of ‘novel’ ecosystems since the 2000s (e.g. Hobbs et al. 2009). While these shifts in social values happen slowly in the order of decades, current climate change concerns and local political movements may be further contributing to this growing emphasis on the values of native species in cities (Kendal and Raymond 2019).

### Implications for urban green space management

As the climates of cities become increasingly hostile to many native species, decision-making around the nativeness of the urban forest is likely to involve conflicts, particularly among those for whom nativeness is a salient issue. Although the findings of this study demonstrate a convergence of attitudes towards native species, there are nevertheless a diversity of values attached to native and non-native urban trees that need to be incorporated into management decisions. Attempting to change people’s values to solely align with biodiversity conservation objectives is unlikely to be a feasible approach (Manfredo et al. 2017), nor consistent with the provisioning of a broader set of ecosystems services, including cultural services (Fish et al. 2016), in highly managed (or ‘novel’) urban settings. Strategies to mitigate the effects of climate change on the urban forest, such as climate-adjusted provenance selection (Breed et al. 2013) or irrigation of urban trees (Doll et al. 2022), need to be considered within the complexity of meanings that people ascribe to native and non-native trees and landscapes, and that these meanings can change with time and changing physical (e.g. climate) and social (e.g. food production) circumstances. This will likely necessitate value trade-offs informed by placed-based research such as ours to ensure that the benefits of nativeness are maintained across the urban landscape.

## Conclusions

We have shown that people’s experiences and values of urban nature are often shaped by nuanced subjective meanings given to the nativeness of trees and landscapes, which do not always align with ecological approaches to urban environmental management. Both native and non-native trees are valued in public urban green spaces, and for a range of reasons and attributes. While our findings point to a convergence of perceptions and importance of native species, nativeness is not equally salient for all people and in all contexts. Therein lies the seeds of conflict around the strategies and actions relating to selecting trees for public urban areas. To meet the needs and preferences of diverse urban publics, and to provide the functions and services they seek, urban greening strategies need to recognise and consider the complex relational values and benefits of nativeness in novel public urban green spaces. With this understanding, tree selection decisions can be driven by multiple ecological and social objectives that can strike a sustainable balance between functions and services that consider both place and people.

## Appendix A: Questions used as conversation prompts in interviews

1. What comes to mind when I say urban green space?
2. How often do you spend time in public urban green spaces?
3. What kinds of public green spaces do you spend time in? Do you tend to visit different kinds of green spaces for different reasons?
4. Are you aware of native and non-native plants in those spaces?
5. What makes a plant native for you? Can you give an example of a native plant?
6. What makes a plant non-native? Can you give an example of a non-native plant?
7. Do native/non-native plants play a role in your reasons for visiting different kinds of urban green spaces?
8. Should native/non-native plants be in cities? If so, where do they belong?
9. Why is it important to you for [use an example UGS mentioned by interviewee] to include native plant species?
10. Why is it important to you for [use an example UGS mentioned by interviewee] to include non-native plant species?
11. What are the benefits of having native/non-native plants in urban green spaces?
12. Are there any problems with having native/non-native plants in urban green spaces?
